# Sleep apnea and severe bradyarrhythmia – an alternative treatment option: a case report

**DOI:** 10.1186/s13256-015-0596-6

**Published:** 2015-05-15

**Authors:** Amin Daoulah, Sara Ocheltree, Salem M Al-Faifi, Waleed Ahmed, Alawi A Alsheikh-Ali, Farhan Asrar, Amir Lotfi

**Affiliations:** 1 Section of Adult Cardiology, Cardiovascular Department, King Faisal Specialist Hospital & Research Center, P.O. Box 40047, Jeddah, 21499 Kingdom of Saudi Arabia; 2 Internal Medicine Department, University of Alabama Huntsville Regional Medical Campus, Huntsville, Alabama USA; 3 Section of Pulmonology, Internal Medicine Department, King Faisal Specialist Hospital & Research Center, Jeddah, Kingdom of Saudi Arabia; 4 Section of Infectious Disease, Internal Medicine Department, King Faisal Specialist Hospital & Research Center, Jeddah, Kingdom of Saudi Arabia; 5 Heart and Vascular Institute, Sheikh Khalifa Medical City, Abu Dhabi, United Arab Emirates; 6 Department of Medicine, Tufts University School of Medicine, Boston, MA USA; 7 Department of Family Medicine, McMaster University, Hamilton, Ontario Canada; 8 Health & Counselling Centre, University of Toronto, Mississauga, Ontario Canada; 9 Baystate Medical Center, Tufts University School of Medicine, Springfield, Massachusetts USA

**Keywords:** Bradycardia, Sinus arrest, Sleep apnea, Theophylline

## Abstract

**Introduction:**

Sinus arrest, atrio-ventricular block, supraventricular, and ventricular arrhythmias have been reported in patients with sleep apnea syndrome. The arrhythmias usually occur during sleep and contribute to the cardiovascular morbidity and mortality, and the treatment of sleep apnea usually results in the resolution of the brady- arrhythmias. Weight loss, continuous positive airway pressure (CPAP), oral appliances, and upper airway surgery are the recommended treatments, however, compliance and efficacy are issues.

**Case presentation:**

A 58-year-old Arab man presented with recurrent presyncope. He was subsequently diagnosed with sleep apnea associated with frequent and significant sinus pauses. He presented a treatment challenge because he refused continuous positive airway pressure and pacemaker, however, he was successfully treated with theophylline.

**Conclusion:**

Frequent and significant sinus pause associated with sleep apnea was successfully treated with theophylline in our patient when the standard treatment of care was refused.

## Introduction

Sleep apnea-associated bradyarrhythmias contribute to the cardiovascular mortality and morbidity of sleep apnea [1-4] and treatment of sleep apnea usually results in resolution of bradyarrhythmias [3]. To date, weight loss, continuous positive airway pressure (CPAP), oral appliances, and upper airway surgery are the recommended treatments; however, compliance and efficacy are issues [5,6]. Our patient presented a treatment challenge because he had significant sinus pauses associated with sleep apnea, but he refused recommended treatment. Theophylline, used to treat brady-arrhythmia in different settings, was introduced as an alternative treatment option and was successful in treating our patient.

## Case presentation

A 58-year-old Arab man was referred to our electrophysiology clinic with monthly episodes of presyncope for the last 3 months. Despite two episodes of presyncope per year for 3 years, he had not sought medical advice. He had multiple comorbidities including coronary artery disease status post coronary artery bypass surgery (CABG), type 2 diabetes mellitus, dyslipidemia, and class III obesity (body mass index 41) with a neck circumference of 45cm and modified Mallampati score of 4.

**Figure 1:**
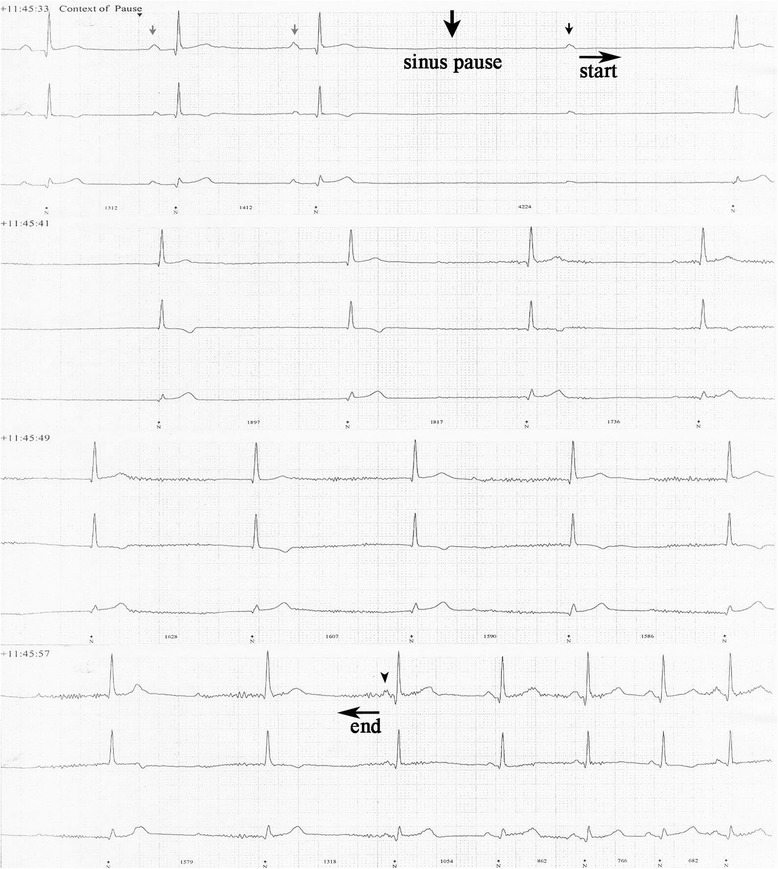
Holter recording displaying a prolonged sinus pause. A sample from the Holter recording displaying a non-conducted P-wave (small black arrow) preceded by a sinus pause of 2.7 seconds (large black arrow) and followed by sinus arrest of 22 seconds with junctional escape beats (between the horizontal start and end arrows). The gray arrows point to sinus bradycardia and black arrowhead points to the first P-wave, after the return of sinus rhythm. Holter revealing a non-conducted P-wave (small black arrow) followed by sinus arrest (horizontal arrows).

After his CABG, aspirin, atorvastatin, and metformin were restarted, with the addition of metoprolol tartrate 25mg twice daily. More frequent episodes of presyncope occurred. An electrocardiogram revealed normal sinus rhythm with normal PR, QRS and QTc intervals. Transthoracic echocardiography demonstrated a normal left ventricular systolic function and 24-hour ambulatory Holter monitoring recorded multiple sinus pauses occurring from 11:22 p.m. until 11:46 a.m. with a maximum pause of 22 seconds occurring at 11:45:33 a.m. (see Figure 1). During this observation, there were no episodes of presyncope.

He was admitted to our hospital for further workup. Despite discontinuation of metoprolol for 1 week, his telemetry revealed multiple episodes of sinus pause, observed during daytime sleepiness or snoring episodes. Obstructive sleep apnea (OSA) was suspected and a sleep study demonstrated both central and OSA: Epworth score 7, sleep latency of 17 minutes, Apnea–Hypopnea Index (AHI) of 98/hour, arousal index of 49/hour and lowest oxygen saturation at 78% on room air (Table 1).

**Table 1:** **Symptoms reported, Holter monitor, theophylline levels and polysomnography results at presentation and during follow up**

	**Symptoms**	**Weight (kg)**	**24-hour Holter monitor**			**Theophylline level** ^*^	**Polysomnography**
			**Max sinus pause (seconds)**	**Total number of sinus pauses**	**Min/max/Average HR (beats/minute)**		
Prior to presentation (2008 until 2011)	Presyncope twice a year for 3 years	115	–	–	–	Not on treatment	–
At presentation (September 2011)	Presyncope once a month for 3 months	119	22	71	49/119/85	Not on treatment	Epworth score 7
							Sleep latency of 17 minutes
							AHI of 98/hour
							Arousal index of 49/hour
							Lowest oxygen saturation at 78% on room air
5-month follow up	None	120	6	18	61/125/93	11.11	Not done
6-month follow up	None	122	5	5	68/124/94	13.0	Not done
7-month follow up	None	126	None	None	71/128/92	14.4	Epworth score of 11
							Sleep latency of 28 minutes
							AHI of 96/hour
							Arousal index of 43/hour
							Lowest oxygen saturation at 78% on room air
14-month follow up	None	123	None	None	69/123/90	11.4	Not done
19-month follow up	None	129	2.9	3	67/115/82	14.6	Epworth score of 11
							Sleep latency of 48.5 minutes
							AHI of 83/hour
							Arousal index of 42.8/hour
							Lowest oxygen saturation at 75% on room air
29-month follow up	None	130	None	None	65/110/81	Not done	Not done

AHI = Apnea–Hypopnea Index; HR = Heart rate; Max = Maximum; Min = Minimum.

^*^Therapeutic level is between 10 and 20mcg/mL in plasma [4].

Overnight CPAP was started and the telemetry showed sinus bradycardia with a minimum heart rate of 30 beats per minute with infrequent pauses of less than 3 seconds. However he refused to continue to use the CPAP upon discharge. In addition, he was offered a pacemaker and he refused. A trial of theophylline 200mg twice daily was initiated and he was discharged from our hospital and encouraged to initiate a weight reduction program.

At follow up, he had no further episode of presyncope with infrequent short or no pauses at therapeutic theophylline levels (Table 1). A follow-up sleep study revealed improvement in the central element of his sleep apnea, however, his AHI did not significantly improve; such results are expected with theophylline therapy (Table 1).

## Discussion

Sleep apnea syndrome has been associated with cardiovascular complications including hypertension [1], heart failure [2] and cardiac arrhythmia [3]. Observational studies have shown that treating sleep apnea syndrome can decrease blood pressure [1], reduce cardiac arrhythmia [3] and decrease cardiovascular mortality [4]. Noninvasive positive pressure ventilation effectively decreases the incidence of sleep apnea-associated arrhythmia [3]; however, not all patients can tolerate it and compliance is an issue [5]. We report the case of a patient who presented with presyncope most likely secondary to sleep apnea-induced brady-arrhythmia. He was started on beta-blockers after his CABG, which could have made his brady-arrhythmia worse and contributed to increasing presyncope. However, while he was an in-patient and off his metoprolol he continued to have significant sinus pause. He did not tolerate CPAP and refused a pacemaker so, as a last resort, theophylline treatment was started and he reported complete resolution of his symptoms. A Holter monitor documented a decrease in both the frequency and duration of sinus pauses. We initiated theophylline based on its effectiveness in treating brady-arrhythmia in the setting of post-cardiac transplant [6] and spinal cord injury [7]. The mechanism of brady-arrhythmia in sleep apnea syndrome is hypothesized to be due to the activation of the diving reflex by hypoxemia and apnea, with reflex activation of the cardiac vagal nerve. This induces severe nocturnal bradyarrhythmias, especially during rapid eye movement sleep [8]. Theophylline is a nonselective phosphodiesterase inhibitor that also competitively blocks adenosine receptors resulting in central nervous system and cardiovascular stimulation. Theophylline also increases the respiratory drive and it has been used in patients with central sleep apnea due to left ventricular dysfunction before CPAP and is still recommended in patients who cannot tolerate CPAP [9]. For OSA, theophylline has been shown to mildly reduce obstructive events but is associated with sleep disruption and therefore it is not recommended [9]. The limitations and side effects of theophylline result from its narrow therapeutic index, traditionally between 10 and 20mcg/mL [4] in plasma. Theophylline is metabolized in the liver through the cytochrome P450 system and consequently is implicated in several drug interactions with commonly prescribed drugs, which may increase serum concentrations of theophylline [10].

## Conclusions

We report a case of treating sleep apnea-induced brady-arrhythmia with theophylline when standard treatment of care was refused. Further studies are needed to determine the safety and efficacy of theophylline for treating sleep apnea-induced bradyarrhythmias to ensure the benefits outweigh the risks.

## Consent

Written informed consent was obtained from the patient for publication of this case report and any accompanying images. A copy of the written consent is available for review by the Editor-in-Chief of this journal.

## Abbreviations

AHI: Apnea–Hypopnea Index
CABG: Coronary artery bypass surgery
CPAP: Continuous positive airway pressure
OSA: Obstructive sleep apnea
